# Epistasis in the receptor-binding domain of contemporary H3N2 viruses that reverted to bind sialylated di-LacNAc repeats

**DOI:** 10.1016/j.celrep.2025.116007

**Published:** 2025-07-17

**Authors:** Ruonan Liang, Francesca Peccati, Niels L.D. Ponse, Elif Uslu, Annelies J.H. de Rooij, Alvin X. Han, Geert-Jan Boons, Luca Unione, Robert P. de Vries

**Affiliations:** 1 Department of Chemical Biology & Drug Discovery, Utrecht Institute for Pharmaceutical Sciences, Utrecht University, Universiteitsweg 99, 3584CG Utrecht, the Netherlands; 2 CICbioGUNE, Basque Research & Technology Alliance (BRTA), Bizkaia Technology Park, Building 800, Bizkaia, 48160 Derio, Spain; 3 Ikerbasque, Basque Foundation for Science, 48013 Bilbao, Spain; 4 Department of Medical Microbiology & Infection Prevention, Amsterdam University Medical Centers, University of Amsterdam, Amsterdam, the Netherlands; 5 Complex Carbohydrate Research Center, University of Georgia, 315 Riverbend Road, Athens, GA 30602, USA; 6 Lead contact

## Abstract

Since their introduction into humans, H3N2 influenza A viruses have evolved continuously to escape immunity through antigenic drift, driven by mutations in and around the receptor-binding site. Recently, these changes resulted in viruses that recognize elongated glycans, which are less abundant in the human respiratory tract, complicating vaccine strain propagation. This study employed ELISA, glycan arrays, tissue staining, flow cytometry, and hemagglutinin (HA) assays to demonstrate the molecular determinants of recent H3N2 viruses that regained recognition of shorter glycans. Mutations Y159N/T160I in contemporary strains replace Y159/T160, weakening receptor binding. However, this is compensated by Y195F in the 190-helix. These findings highlight epistasis across critical residues in the HA receptor-binding site, including the 130-loop, 150-loop, and 190-helix. Interestingly, a positive correlation exists between binding to an asymmetrical N-glycan and binding to human and ferret respiratory tract tissues. These results elucidate the epistatic nature of receptor-binding specificity during influenza A virus H3N2 evolution.

## In brief

Liang et al. shows how contemporary H3N2 viruses reverted to bind sialylated di-LacNAc at the molecular level. Using complex symmetrical and asymmetric N-glycans, major epistatic interactions were revealed.

## Graphical Abstract

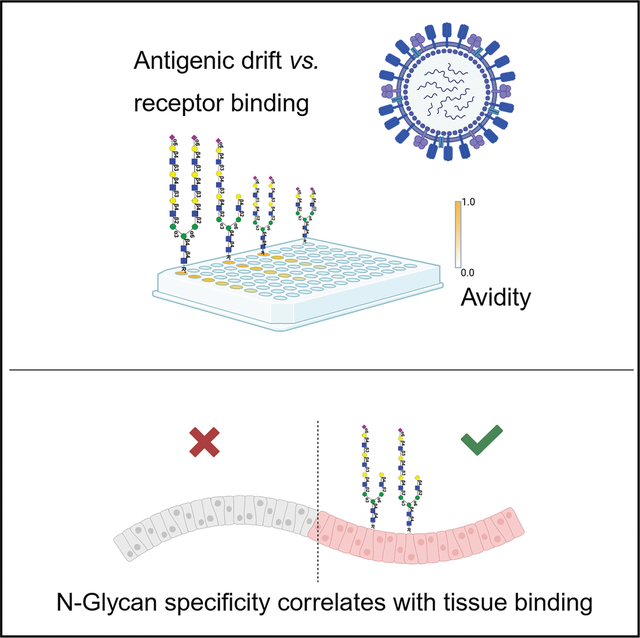

## INTRODUCTION

Influenza A viruses (IAVs), having a reservoir of aquatic birds, occasionally cross the species barrier to infect humans, historically sparking pandemics. IAVs can be divided into 19 subtypes based on the hemagglutinin (HA) and neuraminidase (NA) glycoproteins on the surface of IAVs.^1,2^ HA recognizes sialic acids on the host cell surface and fuses the virus’s membrane with that of the host, while NA promotes the release of new virions from infected cells. Among the HA and NA combinations, three of them, H1N1, H2N2, and H3N2, have been known to circulate in humans for decades. Notably, the introduction of IAVs from avian species to humans coincides with a binding specificity switch from α2,3-linked (avian-type) to α2,6-linked (human-type) sialylated glycans.^3,4^

H3N2 viruses have a remarkable ability to escape host immunity and, as such, have infected the human population during the last six decades. During that time, the virus evaded neutralization by antibodies due to antigenic drift, driven by the accumulation of mutations in the most exposed parts of the globular head of HA. This part of the HA protein contains the receptor-binding site (RBS) composed of four structural elements: the 130-loop, 150-loop, 190-helix, and 220-loop. Thus, antigenic changes in and around the RBS of HAs^5^ avoid antibody neutralization, impact the interactions with sialylated glycans, and, eventually, compromise effective receptor binding.^6–10^ Over the last decade, H3N2 viruses demonstrated altered receptor specificity, resulting in a robust selectivity for branched glycans containing at least three LacNAc repeats.^10–13^ A LacNAc moiety is a disaccharide unit composed of a galactose β4 linked to an N-acetyl-glucosamine. In complex multi-antennary N-glycans, LacNAc units can form repeats of multiple lengths.

Despite the low abundance of LacNAc repeats containing glycans in the human and ferret upper respiratory tract,^14,15^ H3N2 viruses incorporated mutations in the 150-loop that guarantee effective receptor binding to sialylated glycans containing at least 3 LacNAc repeats at the beginning of the current century.^16^ Employing high-resolution structural studies, we and others demonstrated that the aromatic side chain of the HA residue at position 159 was instrumental in the interaction with the innermost LacNAc repeat.^17,18^ There is a glycosylation site at residue 158 (Asn), which is one part of the glycosylation motif 158–160 at antigenic site B. Around 2014–2015, compared with 3C.3a, the new mutation, K160T, in 3C.2a introduced a new glycosylation site and led to a major antigenic change and finally showed a low vaccine efficacy during that season.^19^ Yet, in 2021–2022, the dominating H3N2 strain in the Northern Hemisphere, influenza, had another set of changes at positions 158–160. This antigenic site B now contained Asn, Asn, and Ile at positions 158–160 instead of Asn, Tyr, and Thr (NYT158–160NNI), which causes a loss of a glycosylation through the T160I substitution. This resulted in poor binding to α2,6-linked sialosides in a glycan array format but maintained some binding, specifically to extended branched sialosides.^13^ However, that study did not assess the other HA substitutions in this antigenic drift period, which likely compensated for the weak binding to preserve viral infection. Since January 2020, specific mutations in the 190-helix (G186D and D190N) have coevolved in human H3N2 HAs. A recent contribution from the group of Nicholas Wu demonstrated that the epistasis between G186D and D190N maintains binding to α2,6-sialylated glycans and alters HA antigenicity.^20^ Contemporary strains favor extended glycan receptors, with significant binding to sialylated di-LacNAc structures,^18^ regaining hemagglutination properties critical for antigenic surveillance.^21^ It is, therefore, crucial to understand, at the molecular level, how glycan-binding specificity evolves in contemporary H3N2 influenza viruses.

In this study, we interrogated which HA residues are responsible for the glycan-binding specificity changes during H3N2 evolution in the last decade. We first focused on 3C.3a and 3C.2a1 viruses, the latter split into the 3C.2a.1b.2a.2a.3a.1 clade, revealing the reversion to di-LacNAc specificity.^21^ All current H3N2 viruses contain Y159N/T160I in their HA, which abrogates receptor binding. This diminished receptor binding was rescued by introducing Y195F, present in all currently circulating strains. We demonstrate strong epistasis at amino acids T131K, G186D, D190N, F193S, and Y195F to maintain binding to an asymmetrical α2,6-linked sialic acid tri-LacNAc-containing N-glycan. This and perhaps similar structures appear to be displayed on human and ferret respiratory tract tissues, whereas the symmetrical version is not.

## RESULTS

### Y159 and T160 mutants are responsible for glycan-binding changes during evolution

**Figure 1.**
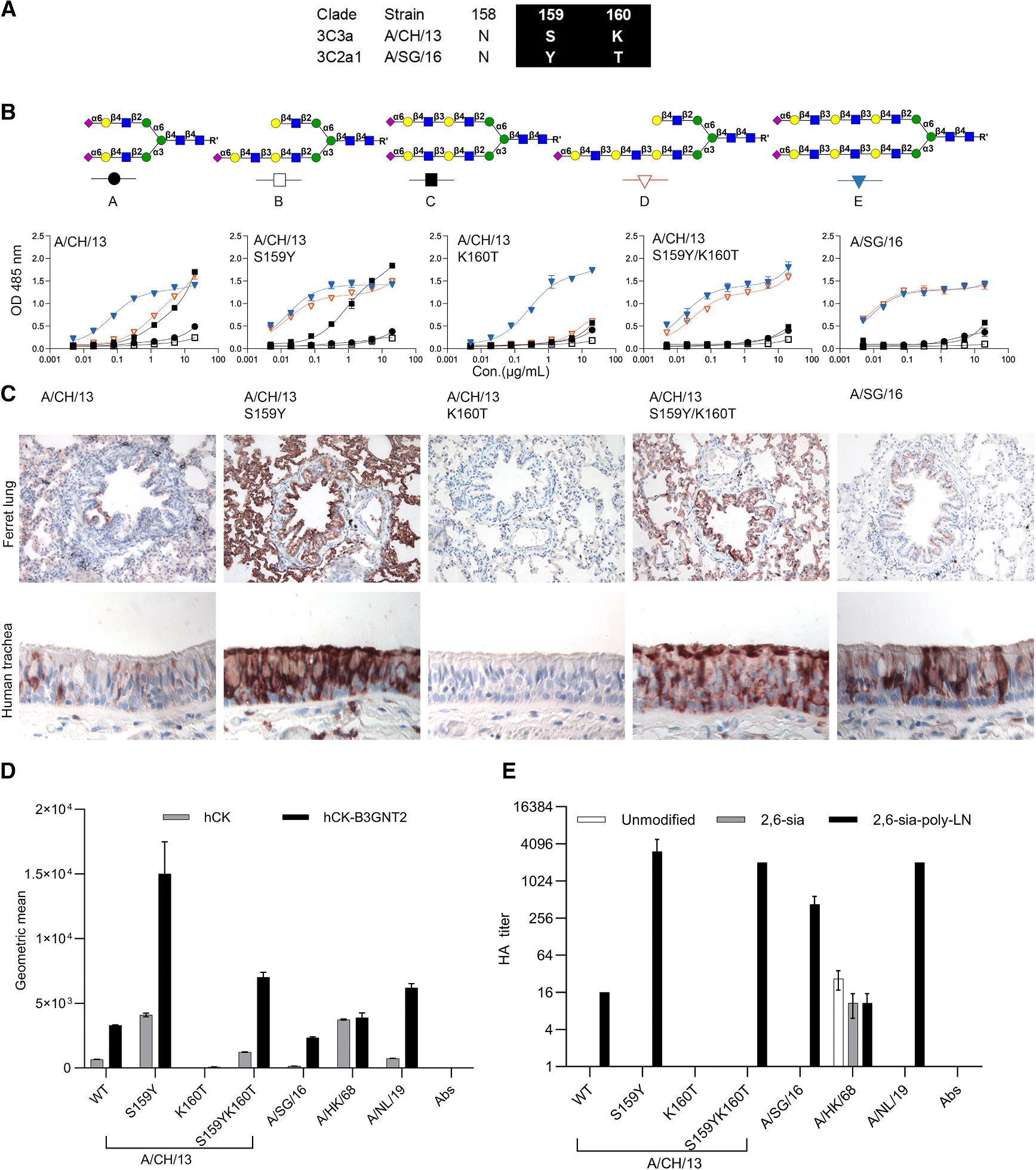
Y159 and T160 are responsible for glycan-binding specificity toward LacNAc (A) Alignment of amino acids 159 and 160 between A/CH/13 and A/SG/16. (B) Glycan structures **A**–**E** and binding avidities of A/CH/13 HAs (WT and mutations) and A/SG/16 to these structures was measured by ELISA. Error bars represent the standard deviation of a duplicate measurement, which represent three biological independent assays. (C) Tissue staining of A/CH/13 HAs (WT and mutations) and A/SG/16 WT to ferret lung and human trachea. (D) Flow cytometry analysis of A/CH/13 HAs (WT and mutations) and A/SG/16 WT to hCK and hCK-B3GNT2 cells. Error bars represent the standard deviation of triplicate measurements, which represent three biological independent assays. (E) Hemagglutination assay of A/CH/13 HAs (WT and mutations) and A/SG/16 WT to WT and glycan-remodified chicken erythrocytes. Error bars represent the standard deviation of triplicate measurements, which represent three biological independent assay.

We and others have recently shown the importance of the Y159 position, as it directly interacts with extended LacNAc structures (Figure 1A).^10,17,18^ These data are derived from glycan arrays and saturation transfer difference (STD) nuclear magnetic resonance (NMR) spectroscopy approaches; more standard affinity measurements, such as direct ELISAs, are lacking. This lack is mainly due to the scarcity of N-glycan material; only minute amounts are necessary for glycan array printing and are, therefore, hardly available for other experimental approaches. Here, we selected several N-glycans terminated with human-type receptors and introduced biotin at the reducing end, enabling a straightforward streptavidin coupling in microtiter plates. All N-glycans terminate with human-type receptors, designated by letters throughout the manuscript (Figure 1B); we generated symmetrical bi-antennary N-glycan with mono-, di-, and tri-LacNAc extensions (**A**, **C**, and **E**) and the asymmetrical counters parts of **C** and **E** in which the α3 arm is extended with either a di-LacNAc (**B**) or tri-LacNAc (**D**) motif. The A/Switzerland/9715293/2013 (A/CH/13) H3N2 virus was part of the vaccine during the 2014–2015 season and was chosen as a representative of the 3C.3a clade; conversely, A/Singapore/INFIMH-16–0019/2016 (A/SG/16), part of the 2018–2019 Northern Hemisphere flu vaccine, was chosen as a representative of the 3C.2a1 strain. A/CH/13 encodes amino acids NSK at positions 158–160, and A/SG/16 encodes NYT at positions 158–160 (Figure 1A).

In our ELISA approaches, we aimed to determine, within a single assay, both specificity and avidity using trimeric HAs. A/CH/13 wild-type (WT) HA preferentially binds the symmetrical tri-LacNAc compound **E** with less avidity to the symmetrical **C** and the asymmetrical tri-LacNAc structure **D** (Figure 1B). A/CH/13 HA containing S159Y demonstrated an increased avidity to **C**–**E** compounds compared to the WT, with the most notable increase in binding to **D**. However, when K160T was introduced, creating an N-linked glycosylation site, we observed a stark decrease in binding, and only compound **E** was bound. Reconstitution of S159Y/K160T based on the A/CH/13 HA backbone, which mimics A/SG/16, showed a similar binding to asymmetrical **D** and symmetrical N-glycan **E**. Interestingly, whereas A/CH/13 WT HA prefers compound **E**, the S159Y/K160T mutant does not differentiate between the symmetrical and asymmetric N-glycans. Quality control was performed using biotinylated *Sambucus nigra* Lectin (SNA), which binds to α2,6-linked sialic acid attached to the terminal galactose, and all compounds containing that structure were similarly bound (Figure S1A). At the same time, a symmetrical N-glycan in which the tri-LacNAc arms were not sialylated and a linear tri-LacNAc compound containing α2,6-linked sialic acid (6′SLN_3_-L) were used as controls (Figure S1C), which showed that only the A/CH/13 S159Y mutant and SNA could bind the linear structure 6′SLN_3_-L. In contrast, all other HA proteins failed to bind to this compound.

Ferret lung and human trachea are biologically relevant respiratory tissues to analyze human IAV receptor-binding properties.^22–25^ A/CH/13 WT HA bound the whole ferret lung weakly, and S159Y significantly increased binding. Conversely, the K160T mutant failed to bind the ferret lung and human tracheal tissue sections. This mutant efficiently bound compound **E** in the ELISA, demonstrating that this structure is extremely rare in tissues. The double mutant S159Y/K160T gained binding to both tissues with less intense signals than the S159Y mutant. The A/SG/16 WT binding signal is restricted to the ferret bronchi, and we hypothesize that this is a biological difference between 3C.3a and 3C.2a1 viruses. These data indicate that introducing an N-glycosylation site is detrimental to receptor binding. At the same time, and as we previously have demonstrated,^17^ the CH-pi interaction with 159Y confers improved binding avidity to tri-LacNAc-containing glycans and, therefore, can bind complex asymmetrical N-glycan **D**. Such interactions are very often observed in glycan-protein complexes and contribute largely to binding.^26^

“Humanized” Madin-Darby canine kidney (hCK) cells solely display glycan structures with α2,6-linked sialic acid,^27^ the key receptors of human IAVs. We and others recently created hCK-B3GNT2 cell lines by overexpressing the B3GNT2 enzyme to increase the number of LacNAc repeat units, having a more human-like glycan profile.^28–30^ The binding ability of the A/CH/13 and A/SG/16 was analyzed for these two cell lines using flow cytometry (Figure 1D). A/Hongkong/1/1968 (A/HK/68) HA was used to detect α2,6-linked sialic acid presented in WT cells, as this protein can bind α2,6-linked sialic acid presented on N-glycans as a mono-LacNAc structure.^29^ Conversely, we used A/Netherlands/00010/2019 (A/NL/19) HA protein as the control, as this protein solely recognizes **E**.^28^ A/CH/13 S159Y exhibited at least three times more binding to hCK-B3GNT2 cells than hCK cells. K160T abrogated binding to hCK-B3GNT2 cells, consistent with the ELISA and tissue staining results. The S159Y/K160T double mutant showed less binding than the S159Y mutant, comparable to the A/SG/16 WT protein, and both exclusively bound to hCK-B3GNT2 cells.

The HA assay is a typical method to monitor IAV receptor binding; however, during antigenic drift, 3C.2a1 viruses have lost the ability to bind turkey erythrocytes.^10^ We created glycoengineered erythrocytes to display N-glycans with elongated arms terminating in α2,6-linked sialic acid (2,6-sia poly-LN). We also made red blood cells that we treated with NA, and after that, α2,6 resialylated; therefore, these cells do not contain elongated LacNAc repeats (2,6-sia).^10^ We used these glycoengineered red blood cells to examine the agglutination titers of the A/CH/13 and A/SG/16 HA WT proteins and their mutants (Figure 1E). As a control, A/HK/68, agglutinated WT, 2,6-sia and 2,6-sia poly-LN erythrocytes, and A/NL/19 only efficiently agglutinated 2,6-sia poly-LN erythrocytes. A/CH/13 WT only agglutinated 2,6-sia poly-LN erythrocytes^10^; importantly, S159Y and S159Y/K160T strongly agglutinated the erythrocytes, having extended sialylated LacNAc moieties (2,6-sia poly-LN erythrocytes), similar to the A/SG/16 WT.

### Positions 159 and 160 are asparagine and isoleucine in currently circulated H3N2 viruses, severely affecting receptor binding

**Figure 2.**
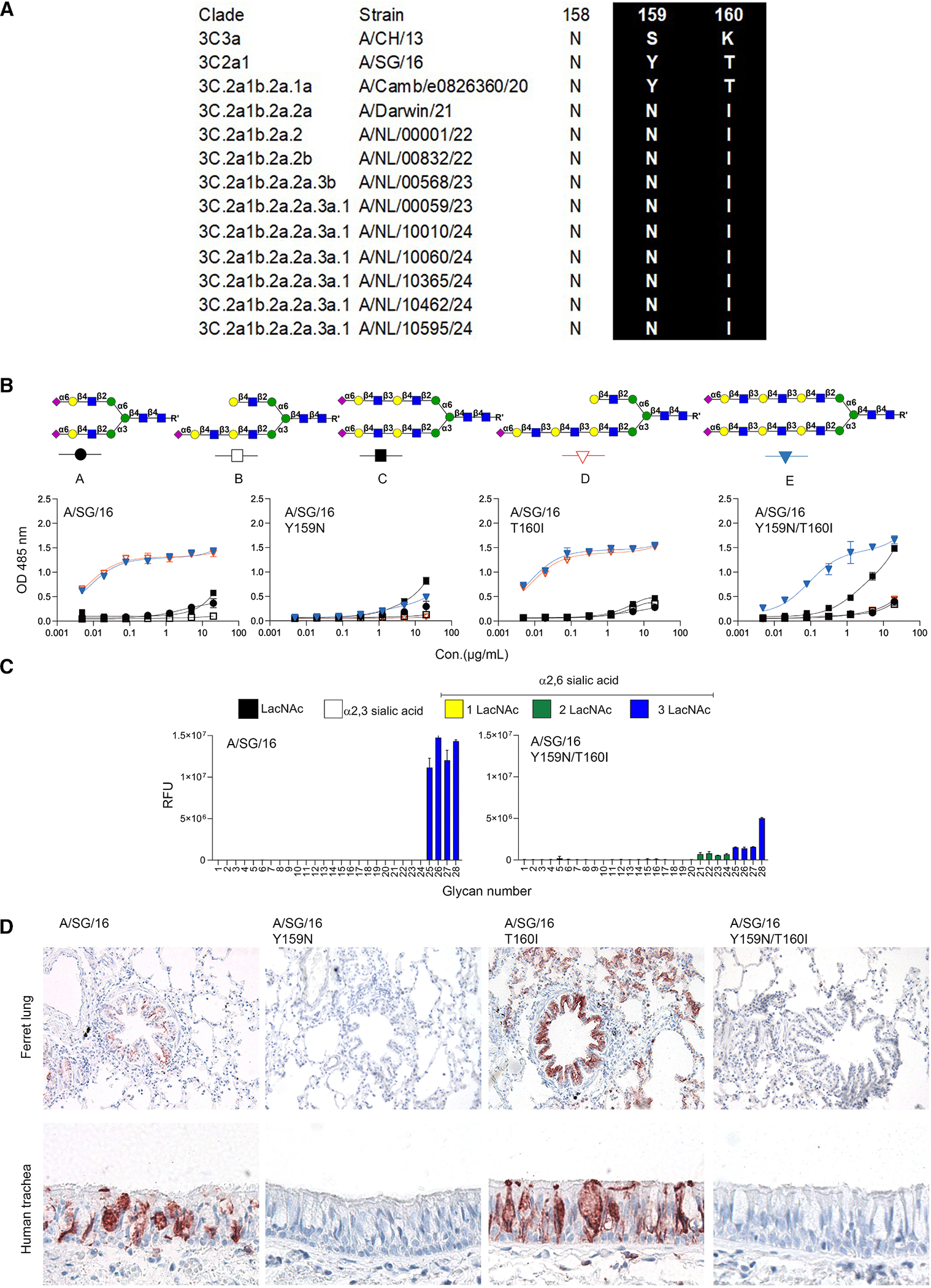
The binding specificities of A/SG/16 HAs (WT and mutations) (A) Alignment of residues at 159 and 160 of 3C.2a.1 and its descendants. (B) Binding avidities of A/SG/16 HAs (WT and mutations) measured by ELISA. Error bars represent the standard deviation of the duplicate measurements, which represent three biological independent assays. (C) Glycan array analysis of A/SG/16 WT and Y159N/T160I. Error bars represent the standard deviation of four replicates on the array and represent two biological independent assays. (D) The tissue staining of the A/SG/16 HAs (WT and mutations) to ferret lung and human trachea.

Currently circulating H3N2 viruses have 22 amino acid mutations in HA1 when we compare the A/Netherlands/10595/2024 sequence with that of A/SG/16 (the complete alignment is shown in Figure S2). Among other amino acid changes in and around the RBS, the changes at positions Y159N and T160I are noteworthy (Figure 2A). They result in the loss of the CH-pi interaction between 159Y and the innermost galactose,^17^ while T160I results in a loss of an N-glycosylation site; both these mutations impact receptor-binding properties.^13^

To analyze the receptor-binding avidity changes to biologically relevant N-glycans, we expressed several mutants based on A/SG/16 HA, including Y159N, T160I, and Y159N/T160I, and employed these proteins in our ELISA approaches. The ELISA binding curves demonstrate that A/SG/16 Y159N abrogates binding to all the tested glycans compared to the WT, explaining the instrumental role of Y159 for efficient receptor binding (Figure 2B). Instead, the T160I mutant exhibited a near-identical binding profile compared to the WT by ELISA. Finally, the double mutant Y159N/T160I is bound efficiently to compound **E** and weakly to compound **C**.

We also analyzed the binding specificity of A/SG/16 and A/SG/16 Y159N/T160I using a glycan array^21^ (Figure S3). A/SG/16 solely bound to N-glycans containing tri-LacNAc repeats, whereas A/SG/16 Y159N/T160I was also able to bind di-LacNAc structures, albeit with quite low responsiveness (Figure 2C).

Next, we analyzed binding to ferret lung and human trachea tissue slides using the same A/SG/16 WT and mutant HA proteins. A/SG/16 WT bound to the bronchi in the ferret lung and goblet cells in the human trachea (Figure 2D). The Y159N mutant failed to bind both tissues, as expected. Interestingly, the T160I binding profile significantly differed from the WT, as it could bind the alveoli in the ferret lung and bound in the epithelial layer of the human trachea. This might be due to the loss of a glycosylation site, making room to bind additional complex N-glycans. Y159N/T160I, on the other hand, completely failed to bind to both tissues, while it did bind to compound **E**, indicating that this compound is hardly displayed in these tissues.

### F195 can rescue the binding profile of A/SG/16 Y159N/T160I

**Figure 3.**
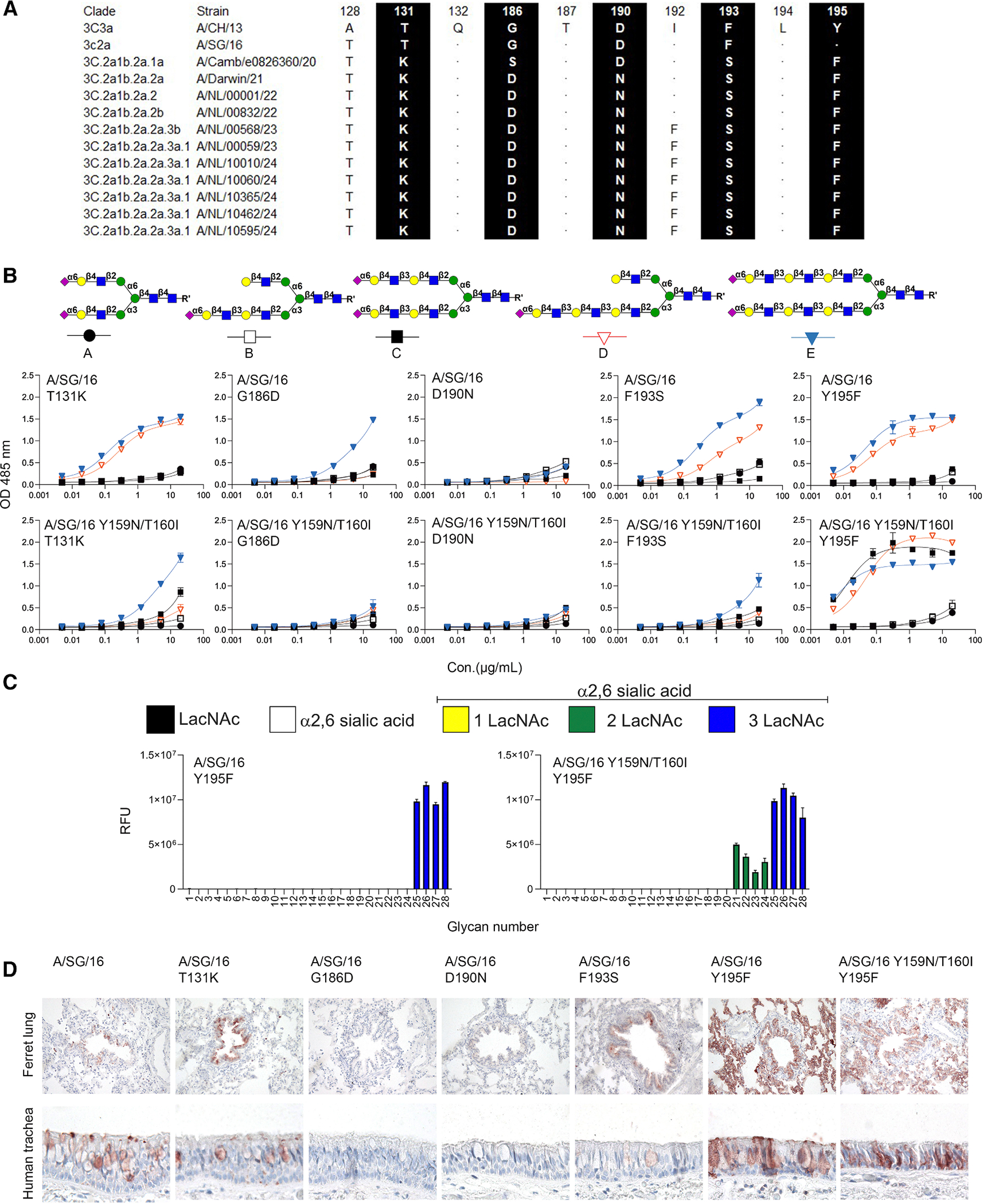
Y195F rescues abrogated binding due to Y159N/T160I (A) Alignment of the residues in 130-loop and 190-helix of H3N2 3C.2a1 viruses. (B) Binding avidities of A/SG/16 and A/SG/16 Y159N/T160I mutant H3 proteins. Error bars represent the standard deviation of the duplicate measurements, which represent three biological independent assays. (C) Glycan array analysis of A/SG/16 Y195F and Y159N/T160I/Y195F. Error bars represent the standard deviation of four replicates and represent two biological independent assays. (D) Tissue staining of A/SG/16 HAs (WT and mutations).

Next to Y159N and T160I, several other amino acid changes lie directly in the RBS, which became fixed in currently circulating H3N2 viruses. These include T131K, G186D, D190N, F193S, and Y195F (Figure 3A). It is unknown how T131K would affect receptor-binding properties, but it is relatively conserved in human influenza viruses (45% of all H1, H2, and H3^31^). Amino acids at 186,^20,32^ 190,^5,33^ and 193,^3,34,35^ on the other hand, are responsible for binding to LacNAc moieties in H3 and other subtypes. Finally, Y195 is extremely conserved (Figure S4) throughout all IAV subtypes and is considered part of the base of the RBS with Y95, W153, H183, and L194.^36^ To analyze their effect on receptor binding in contemporary H3N2 HA, we made single-point mutants (T131K, G186D, D190N, F193S, and Y195F) in A/SG/16 and in the A/SG/16 Y159N/T160I background. The ELISA results demonstrate that T131K, F193S, and Y195F bound to compounds **D** and **E**. G186D exhibited weak binding to **E**, and D190N failed to bind at all (Figure 3B). When the mutants were based on Y159N/T160I, the G186D, D190N, and F193S mutants lost binding to all compounds, and the Y159N/T160I/T131K mutant bound with less avidity compared to the A/SG/16 T131K mutant. Surprisingly, Y159N/T160I/Y195F bound with very high avidity to compounds **C**–**E**; thus, the abrogation of tissue binding by A/SG/16 due to Y159N/T160I is rescued by Y195F.

To further characterize the receptor-binding specificities converted by the Y195F, glycan arrays were performed. We demonstrate that Y195F without Y159N/T160I is only able to bind N-glycans carrying tri-LacNAc motifs. Only in the background of Y159N/T160I, Y195F gains binding to N-glycans displaying di-LacNAc structures (Figure 3C). Based on the results (Figure 2C), it is possible that the gain of di-LacNAc structures is due to N159/I160, but the binding is strengthened by Y195F. Thus, the gain of binding avidity by Y195F only occurs when the 150-loop contains N159 and I160.

**Figure 4.**
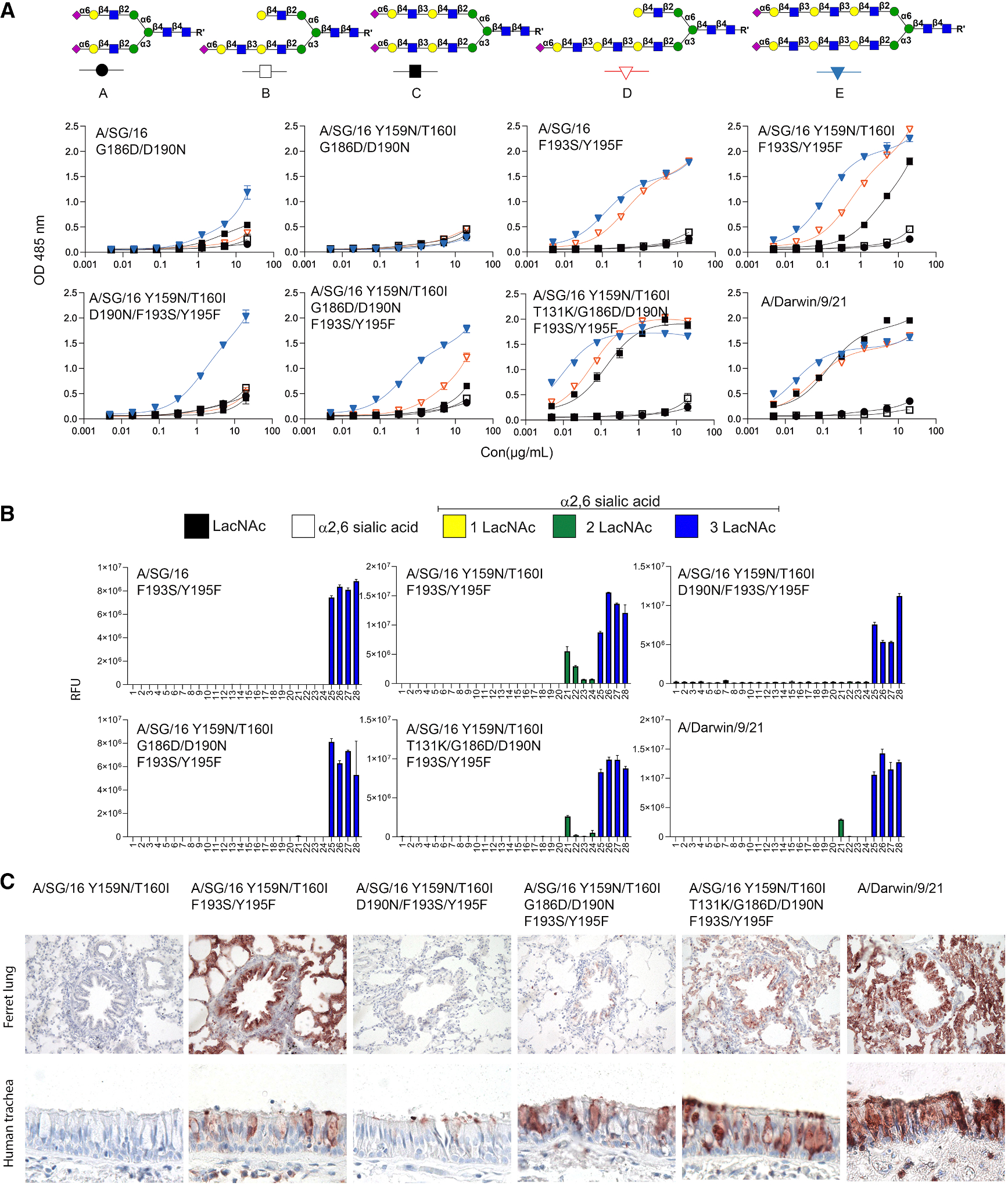
The effect of residues 131, 186, 190, 193, and 195 on receptor binding (A) Binding avidities of different mutational combinations. Error bars represent the standard deviation of the duplicate measurement, which represent three biological independent assays. (B) Glycan array analysis of H3 A/SG16 Y195F mutants. Error bars represent the standard deviation of four replicates and represent two biological independent assays. (C) Tissue staining of the same HA proteins.

Additional flow cytometry analyses using hCK-B3GNT2 cells demonstrate that single mutants T131K, F193S, and Y195F are biologically active, while G186D and D190N are not (Figure S5). In the background of Y159N/T160I, only the Y195F can bind hCK-B3GNT2 cells, further confirming the unique gain of binding afforded by Y195F. Interestingly, the single Y195F mutant bound these cells with higher avidity compared to the Y159N/T160I/Y195F mutant, contradicting the data shown in Figure 4B. However, these cells specifically display elongated LacNAc repeats, and an increase in binding to di-LacNAc structures might be obscured.

To connect binding compounds **D** and **E** to ferret lung and human tracheal cells, we conducted a tissue staining experiment using a selection of mutants: T131K, G186D, D190N, F193S, Y195F, and Y159N/T160I/Y195F (Figure 3D). A/SG/16 WT, T131K, and F193S exhibited binding in the bronchi of the ferret lung and human trachea, whereas the G186D and D190N mutants did not. Y195F and Y159N/T160I/Y195F strongly bind in the whole ferret lung and human trachea. Conclusively, Y195F rescues the detrimental effect of Y159N/T160I on receptor binding.

### A case of differential effects on receptor binding of amino acids in the 130- and 150-loops and the 190-helix

To examine the functional interactions among the mutations, we constructed different constellations and assessed their binding activities. We created double mutations G186D/D190N and F193S/Y195F with or without Y159N/T160I and assembled tri-, tetra-, and penta-mutations in the Y159N/T160I background. The ELISA analysis demonstrated that Y159N/T160I did not affect the abrogation of binding due to G186D/D190N, as hardly any binding was observed (Figure 4A). F193S/Y195F and Y159N/T160I/F193S/Y195F showed strong binding to compounds **D** and **E**; the latter could also bind to **C**. The Y159N/T160I/D190N/F193S/Y195F mutant weakly bound to **E**, and binding was hardly improved with the introduction of G186D, perhaps slightly to compound **D**. With the additional introduction of T131K, resulting in the Y159N/T160I/T131K/G186D/D190N//F193S/Y195F (7-mutant) displaying strong binding avidity to compounds **C**–**E**. Since the World Health Organization recommended that vaccines for use in the 2023–2024 Northern Hemisphere influenza season include the A/Darwin/9/2021 (H3N2) strain, we used it as a control of the dominating 3C.2a.1b.2a.2a.3a.1 strain. The binding activity of A/Darwin/9/21 was almost identical to that of the 7-mutant. We additionally tested several combinations of mutations to compare receptor-binding profiles; we noticed, however, that Y195F had a negative impact on protein expression (Figure S6A). The D190N/G186D/T131K was only able to bind symmetrical compound **E**, and D190N/G186D/T131K/Y159N/T160I did not bind any compound (Figure S6B). The A/SG/16 D190N/G186D/T131K/Y195F HA protein, on the other hand, demonstrated a decreased binding avidity to **D** and no binding to **C** compared to A/Darwin/9/21 (Figure S6B).

We wondered which of these mutants would convert binding to N-glycans with other asymmetrical N-glycans with di-LacNAc motifs, as none of the proteins tested recognizes compound **B**. We thus performed glycan array analysis in which we demonstrated that F193S/Y195F is only bound to N-glycans with tri-LacNAc structures. While the Y195F mutant in the background of Y159N/T160I confers binding to N-glycans with di-LacNAc motifs (Figure 3C), adding D190N (Y159N/T160I/D190N/F193S/Y195F) into this background resulted in a loss of di-LacNAc binding, which was not regained by adding G186D (Y159N/T160I/G186D D190N/F193S/Y195F). As with the ELISA data, the additional introduction of T131KY159N/T160I/T131K/G186D/D190N/F193S/Y195F exerted binding to a di-LacNAc motif, and binding was nearly identical to that of A/Darwin/9/21. Remarkably, compound **21** was preferred over other di-LacNAc-containing structures. The difference in structures **22–24** is the additional introduction of LacNAc on the α6 arm (Figure S3), probably creating steric hindrance.

Finally, we tested the mutants for binding to ferret lung and human trachea tissues. F193S/Y195F converts binding in the Y159N/T160I background, but D190N/F193S/Y195F and G186D/D190N/F193S/Y195F hardly or weakly bound epithelial tissues (Figure 4C). The latter, interestingly, only demonstrated binding to human tracheal tissues, which probably correlates to the ability of this protein to bind to **D** and some N-glycans with a di-LacNAc arm (**#21**). When T131K was introduced, the resulting T131K/G186D/D190N/F193S/Y195F mutant bound with high responsiveness to ferret lung and human trachea tissues, nearly identical to A/Darwin/9/21. The supporting data demonstrated that no proteins tested bound unsialylated or a linear tri-LacNAc compound (Figure S7A). Flow cytometry analysis using both hCK and hCK-B3GNT2 cells revealed an overall preference for hCK-B3GNT2 cells in which we confirmed the interaction between Y159N/T160I and G186D/D190N, which are detrimental for receptor binding, with T131K and Y195F rescuing binding. The 7-mutant has nearly identical receptor-binding properties to A/Darwin/9/21 (Figure S7B).

### The structural ramifications of the Y159N/T160I and Y195F mutations revealed by molecular modeling

**Figure 5.**
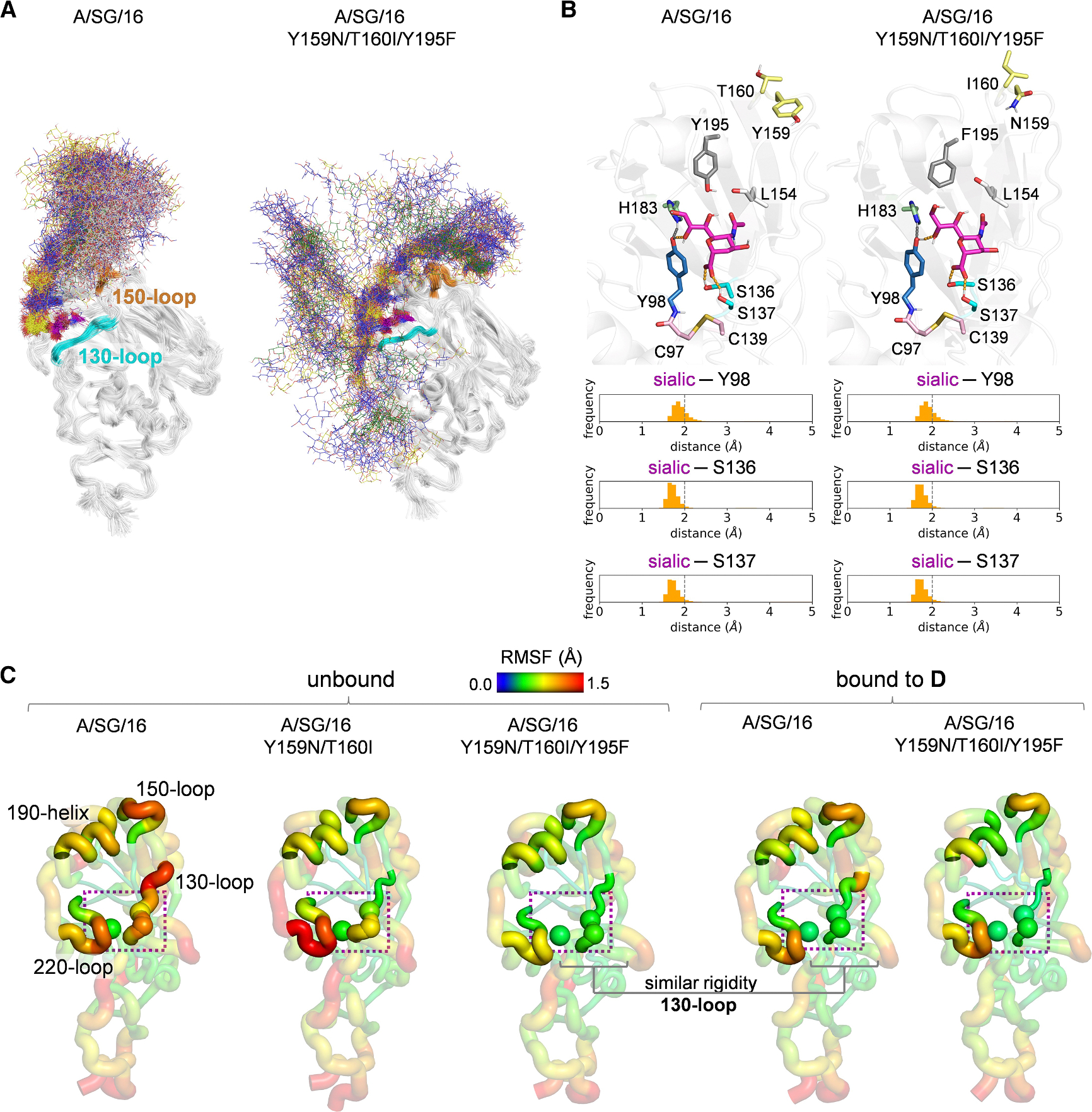
The Y159N, T160I, and Y195F mutations significantly reconfigure receptor-binding site flexibility (A) Overlay of 120 frames sampled with an even stride from three 400 ns MD replicas (1.2 μs total simulation time) of A/SG/16 and A/SG/16 Y159N/T160I/Y195F HA monomeric RBS models bound to compound **D**. **D** is shown as colored lines and the N158 glycan as white lines. The 130-loop and 150-loop are highlighted in cyan and orange, respectively. (B) Distribution of hydrogen bond distances between the sialic acid of **D** and the side chains of Y98 and of S136 and S137 along the same MD simulations. Relevant distances are shown as orange dashed lines. C97, C139, L154, Y/N159, T/I160, H183, and Y/F195 are shown as sticks. (C) Protein backbone root-mean-square fluctuations (RMSFs) along the MD simulations (1.2 μs) of unbound HA monomeric RBS models of A/SG/16, A/SG/16 Y159N/T160I, and A/SG/16 Y159N/T160I/Y195F and the same models of A/SG/16 and A/SG/16 Y159N/T160I/Y195F bound to **D**. The 130-loop, 150-loop, 190-helix, and 220-loop are highlighted. Cα atoms of Y98, S136, and S137 are shown as spheres. **D** and the N158 glycan are not shown for clarity.

Y159N and T160I have apparent structural effects on receptor binding, including the loss of the CH-pi interaction with the innermost galactose of tri-LacNAc-containing glycans and the loss of a glycosylation site. Y195 is exceptionally conserved in all IAVs and forms the base of the RBS with Y98 and H183. To characterize how these mutations impact receptor binding at the molecular level, we performed all-atom microsecond molecular dynamics (MD) simulations of monomeric models of the HA RBS of A/SG/16 and A/SG/16 Y159N/T160I/Y195F bound to **D**. Figure 5A shows an overlay of 120 MD frames, illustrating the flexibility of the bound compound **D**. In A/SG/16, the CH-pi interaction between the aromatic ring of the Y159 and the innermost Gal engages the extended chain of **D** in close contact with the 150-loop. Instead, in the Y159N/T160I/Y195F mutant, the loss of the CH-pi interaction, together with the lack of the glycosylation site at N158, ablates the intermolecular contacts between **D** and the 150-loop, suggesting a marginal role of this loop in binding **D**. Yet, at the sialic acid binding site, the HA-receptor interactions are preserved. Critical contacts for sialic acid binding include a hydrogen bond between O8, the glycerol chain of sialic acid, the side chain of Y98, and a pair of hydrogen bonds between the sialic carboxylate group and the side chains of S136 and S137, located in the 130-loop (Figure 5B). These contacts are preserved along the entirety of the simulations and show equivalent distance distributions in A/SG/16 and the Y159N/T160I/Y195F mutant.

To elucidate the molecular mechanism by which the Y195F mutation rescues binding in the context of the Y159N/T160I mutant, we performed MD simulations of unbound A/SG/16, Y159N/T160I, and Y159N/T160I/Y195F. Our analysis focused on characterizing the flexibility of the four RBS elements—the 130-loop, 150-loop, 190-helix, and 220-loop—and how their conformational dynamics respond to these mutations.

The root-mean-square fluctuation (RMSF) values from the MD simulations for unbound A/SG/16, Y159N/T160I, and Y159N/T160I/Y195F, as well as for the A/SG/16 and Y159N/T160I/Y195F models bound to **D**, are presented in Figure 5C. The Y159N/T160I mutant in the unbound state exhibits significant changes in the flexibility of the RBS elements relative to A/SG/16, suggesting that this pair of mutations, along with the loss of the N158 glycan, substantially impacts the protein’s dynamic behavior. The addition of the Y195F mutation to the Y159N/T160I variant further tunes RBS flexibility, specifically enhancing the rigidity of the 130-loop. RMSF values of residues S136 and S137 in the unbound Y159N/T160I/Y195F mutant are comparable to those of A/SG/16 and Y159N/T160I/Y195F bound to **D**, suggesting that the Y195F allosterically increases the preorganization of the 130-loop, which is directly involved in sialic acid binding.

**Figure 6.**
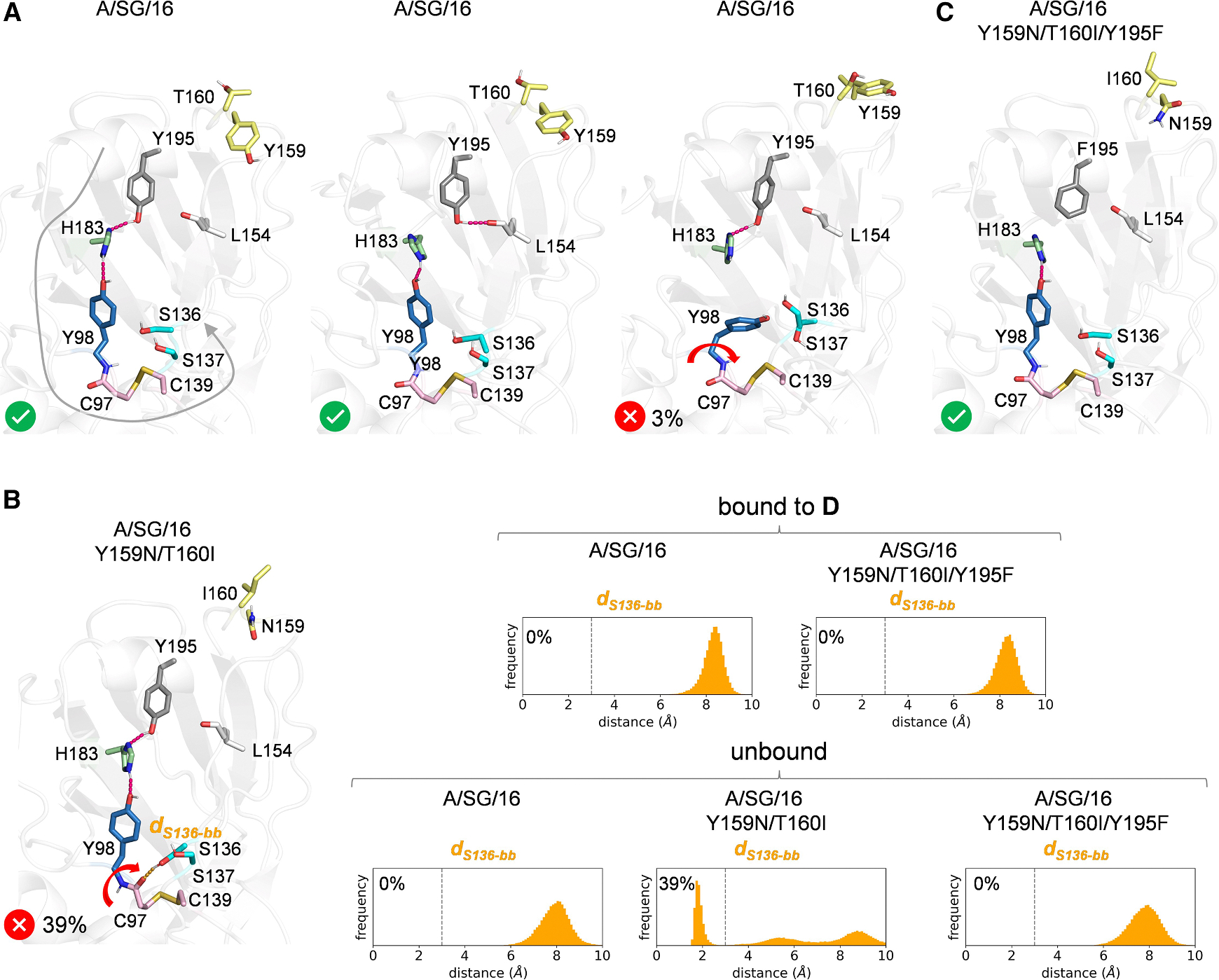
The Y195F mutation allosterically rescues A/SG/16 Y159N/T160I receptor binding (A) Selected frames from three 400 ns MD replicas (1.2 μs total simulation time) of the A/SG/16 HA monomeric RBS model illustrate the allosteric network linking Y195 to Y98 and the 130-loop containing S136 and S137, the alternative hydrogen bond interactions of Y195, and the occasional shift in Y98 rotameric preference, resulting in a conformation not competent for sialic acid binding (3% of the simulation). The gray arrow indicates the extent of the allosteric network that enables communication between RBS elements. The Y98 conformational change is marked with a red arrow. C97, Y98, S136, S137, C139, L154, Y159, T160, H183, and Y195 are shown as sticks. (B) Left: a selected frame from three 400 ns MD replicas (1.2 μs total simulation time) of the Y159N/T160I HA monomeric RBS model, illustrating the alternative backbone-flipped conformation in which the side chain of S136 forms a hydrogen bond with the backbone carbonyl of C97, making it unavailable for sialic acid binding (39% of the simulation). The backbone conformational change is marked with a red arrow. C97, Y98, S136, S137, C139, L154, N159, I160, H183, and Y195 are shown as sticks. Right: distribution of the hydrogen bond distance between the hydroxyl group of S136 and the backbone carbonyl of C97 in the MD simulations of the unbound forms of A/SG/16, Y159N/T160I, and Y159N/T160I/Y195F, as well as the **D**-bound forms of A/SG/16 and Y159N/T160I/Y195F. A vertical line at 3 Å marks the threshold used to quantify the population of the alternative conformation. (C) A selected frame from three 400 ns MD replicas (1.2 μs total simulation time) of the Y159N/T160I/Y195F HA monomeric RBS model showing that H183 is in close contact with Y98. C97, Y98, S136, S137, C139, L154, N159, I160, H183, and F195 are shown as sticks.

Analysis of the MD trajectories identified an allosteric network connecting Y195 with the key residues Y98, S136, and S137 involved in sialic acid binding. Figure 6A shows selected frames from the dynamics simulation of unbound A/SG/16, illustrating this allosteric network. Y195 can alternatively engage in hydrogen bonding with either the backbone carbonyl of L154 at the base of the 150-loop or the Nδ of H183. In turn, H183 forms a hydrogen bond with Y98, preorganizing it for sialic acid binding. The residue immediately preceding Y98, C97, forms a disulfide bridge with C139, which is located on the 130-loop and is in close proximity to S136 and S137. Consequently, the regulation of Y98 flexibility by Y195 through the H183 hydrogen bond is transmitted to the 130-loop via the disulfide bridge. This results in a high level of backbone and side-chain preorganization in A/SG/16, with only 3% of the MD frames showing the Y98 side chain rotating away from H183, adopting a conformation that is not preorganized for sialic acid binding (Figure 6A). Therefore, Y195 provides allosteric communication between the 150-loop and the region encompassing Y98 and the 130-loop, both involved in recognizing tri-LacNAc structures.

The Y159N/T160I variant exhibits markedly different behavior due to the flexibility changes induced by the mutations. Specifically, only in this variant do we observe a backbone flip involving the peptide bond between C97 and Y98 (Figure 6B). This backbone flip exposes the carbonyl oxygen of C97 for interaction with the 130-loop and repositions the side chain of S136, allowing it to form a hydrogen bond with the backbone. As a result, when this alternative conformation is populated, S136 is no longer available for sialic acid binding. Combined with the loss of the CH-pi interaction due to the Y159N mutation, these factors are likely the primary contributors to the loss of binding observed in the double mutant. The population of this alternative, nonbinding-competent conformation was computed in the MD simulations of unbound A/SG/16, Y159N/T160I, and Y159N/T160I/Y195F, as well as the **D**-bound forms of A/SG/16 and Y159N/T160I/Y195F. This was done by monitoring the frequency of the hydrogen bond between the side chain of S136 and the backbone of C97 using a cutoff distance of 3 Å (Figure 6B). While this hydrogen bond is absent in both unbound and **D**-bound A/SG/16 and Y159N/T160I/Y195F, in unbound Y159N/T160I, S136 interacts with the backbone and is therefore unavailable for sialic acid binding in 39% of the simulation.

The Y195F mutation restores binding capacity by rewiring the allosteric network that preorganizes Y98, S136, and S137. In the Y159N/T160I/Y195F mutant, the loss of hydrogen-bonding ability at residue 195 ensures that H183 interacts exclusively with Y98 throughout the entire simulation (Figure 6C), explaining the increased rigidity and preorganization of Y159N/T160I/Y195F compared to A/SG/16 and Y159N/T160I, as reflected in the RMSF values. As a result, the alternative backbone-flipped conformation is eliminated, recovering binding capacity. Therefore, the ability of the Y159N/T160I/Y195F mutant to recognize both di- and tri-LacNAc structures is attributed to its higher affinity compared to A/SG/16, which enhances binding for the common sialic acid unit.

## DISCUSSION

H3N2 viruses continuously evolve to escape population immunity, and this process of antigenic drift occurs due to mutations in and around the RBS.^5,37^ Such mutations block dominant antibodies that neutralize the virus by inhibiting IAV sialic acid binding.^38^ During decades of antigenic drift, human H3N2 viruses have acquired an exquisite receptor-binding specificity for N-glycans carrying an α2,6-sialylated tri-LacNAc at the α3 arm.^10,17^ During the last couple of years, however, these viruses reverted to bind di-LacNAc structures again, which coincides with hemagglutination properties, essential for antigenic surveillance.^21^ Here, we determined the molecular determinants for this reversion of receptor binding and, to our surprise, found that a mutation of a highly conserved Y195 to an F resulted in di-LacNAc binding if the 150-loop contained N159 and I160. Conversely, positions 159 and 160 were vital in the receptor-binding switch between 3C.3 and 3C.2a viruses and their descendants.

The conservation of amino acid in H3 and the other subtype position, 195, were analyzed. Around 2020, the substitution Y195F was introduced in H3 (Figure S3A). However, residue 195 is exceptionally conserved in all other subtypes (Figure S3B) except for H3, which made this substitution so intriguing in the 3C.2a.1b.2a.2a.3a.1. strains. Position 195 is part of antigenic site B and includes residues 159, 160, 186, 190, and 193, all of which independently affect receptor binding. Residue 131, on the other hand, lies in antigenic site A and also affects receptor binding. Single mutations at positions 186 and 190 that abrogate binding are epistatic, as previously shown,^20^ while other single mutants hardly affect receptor binding. The only mutant able to rescue the binding of the Y159N/T160I change was Y195F, emphasizing the importance of this conserved residue.

The residue at 194 is equally conserved and has been shown to undergo egg-adaptive mutations in vaccines, which are, therefore, less effective.^32,39,40^ The epistatic nature of positions 194, 186, and 190 has been demonstrated, as they are highly antigenic.^20^ Interestingly, these HA proteins only bind N-glycans carrying an α2,6-sialylated tri-LacNAc at the α3 arm. Here, we demonstrate that in nature, these viruses revert to binding di-LacNAc structures, as these are presumably more abundant in the human upper respiratory tract.^15^ Interestingly, while H3N2 antigenic drift forced the virus to bind low-abundance complex N-glycans, these viruses did gain polymerase activity.^41^ Thus, following naturally occurring mutations informs us of possible convergent and preferred receptor-binding profiles. It has recently been shown that human H1N1 viruses evolved to bind near identical glycan structures.^21,42^

Our use of complex symmetrical and asymmetric N-glycans displaying either two or three LacNAc repeats at the α3 arm in ELISA-based assays is a first. We employed such structures because viruses equally bound compounds **D** and **E** in glycan arrays. Previously, it was postulated that the symmetrical structures should be preferred since they could engage in a bidentate manner^11^; however, with our recent studies, we clearly showed that the asymmetrical variant is often bound equally well. However, direct avidity differences have not been measured until now. Although our ELISA-based assays are still dependent on multivalency and thus will not resolve a K_D_, we observed significant differences in binding avidity to these structures.

Interestingly, HA proteins that bound the asymmetrical compound **D** could also bind ferret and human respiratory tissues, while proteins restricted to the symmetrical structure **E** did not (Figure S8). We thus like to hypothesize that both human H1N1 and H3N2 viruses preferentially evolved to bind di- and tri-LacNAc structures on the α3 arm, while the α6 is not extended, as it is these kinds of structures that are more abundant in the upper respiratory tract of humans.

These findings provide insights into the complex interplay of mutations affecting glycan binding during IAV evolution at the molecular level. Our data will aid in the functional and antigenic surveillance of ever-drifting H3N2 viruses and assist in picking future vaccine strains.

### Limitations of this study

We attempted to demonstrate the epistatic effects of Y159N/T160I and G186D/D190N with and without T131K/Y195F. However, the Y195F mutation negatively affects protein yields (Figure S6A), and we could not finish a systematic comparison. Additional structural studies, such as X-ray or cryoelectron microscopy (cryo-EM), could reveal how the LacNAc structure interacts with the evolving viruses. Finally, all mutations were created in the HA protein, and how these mutations would influence virus stability or cell entry is not known.

## RESOURCE AVAILABILITY

### Lead contact

Further information and requests for reagents should be directed to and will be fulfilled by the lead contact, Robert P. de Vries (r.vries@uu.nl).

### Materials availability

All unique/stable materials generated in this study are available from the lead contact with a completed materials transfer agreement.

### Data and code availability

Data reported in this paper will be shared by the lead contact upon request. This paper does not report original code.

## STAR★METHODS

### EXPERIMENTAL MODEL AND STUDY PARTICIPANT DETAILS

#### Erythrocytes

All erythrocytes were obtained from adult brown layer chickens (bird species) that are used in the educational program of the Faculty of Veterinary Medicine, Utrecht University, the Netherlands. No sex and gender influence on the results have been observed in our studies.

#### Cell lines

HEK293S GnTI(−), hCK, and hCK-B3GNT2 cells have been authenticated. And all cell lines were tested for mycoplasma contamination.

#### Tissues

Sections of formalin-fixed, paraffin-embedded Ferret (Mustelidae familiaris) lung and Human trachea were obtained from the Department of Veterinary Pathobiology, Faculty of Veterinary Medicine, Utrecht University, the Netherlands and University Medicine Center, Utrecht, the Netherlands (UMCU), respectively. There are no sex and gender influence of the study. The human trachea tissue used in this study was obtained under a Service Level Agreement (SLA2022–006) with UMCU. The sample size is around 0.5 cm * 1 cm * 1.5 cm.

### METHOD DETAILS

#### HA expression and purification

HA encoding cDNAs, A/CH/13, A/SG/16, and A/Darwin/9/21 (synthesized and codon-optimized by GenScript) were cloned into pCD5 expression vector, with a signal sequence, GCN4 trimerization motif, a TEV cleavage site, the twin-strep (IBA, Germany) and a super folder GFP or mOrange2.^45^ Mutations were introduced into HA genes by using site-directed mutagenesis. The HAs were expressed in the HEK 293S GnTI(−) by transfecting the expression vectors with polyethyleneimine I (PEI) (DNA:PEI is 1:8). After 6 h of post-transfection, the transfection medium was moved and replaced by the 293 SFM II expression medium (Gibco), supplemented with 3.0 g/L Primatone (Kerry), 2.0 g/L glucose, 3.6 g/L bicarbonate, 0.4 g/L valproic acid, 1% glutaMAX (Gibco), and 1.5% DMSO. After 5–6 days, the Culture supernatants were collected. HAs were purified by the Strep-Tactin Sepharose beads (IBA, Germany) according to the manufacturer’s instruments.

#### Enzyme-linked immunosorbent assay (ELISA)

Nunc Maxisorp 96 wells plates (Invitrogen) were coated with 50 μL of 5 μg/mL streptavidin (Westburg) in PBS overnight at 4°C followed by the blocking with 300 μL of 1% BSA in PBS-T for 3 hours at room temperature. The streptavidin-coated plates were coated with 50 μL of 50 nM biotinylated glycans overnight at 4°C, followed by the blocking with BSA again. HAs at 20 μg/mL were precomplexed with strepmab and goat-*anti*-human antibodies (Invitrogen (#31410)) in a 1:0.65:0.325 molar ratio on ice for 30 min. Proteins were added to the plates, diluted serially 1:3, and incubated for 2 hours at RT. HA binding was developed by OPD and stopped by the 2.5M H_2_SO_4_ after 5 min. The UV reader (Polar Star Omega, BMG Labtech) measured the optical density at 485 nm. The data was calculated from three independent experiments in duplicate.

#### Glycan microarray binding of HA proteins

The glycan array microarray was performed as described previously.^10,43^ HAs were precomplexed with human anti-streptag-HRP and goat anti-human-Alexa 555 antibodies in a 4:2:1 molar ratio in 40 μL of PBS-T on ice for 30 min. Subsequently, they were incubated on the glycan array surface in a humidified chamber for 90 min. Then, the slides were washed in PBS-T, PBS, and deionized water. After the water had been removed by centrifugation, the slides were immediately scanned. The analysis for processing glycan array data was performed by the script for carbohydrate-microarray processing. After removing the highest and lowest values, the data was calculated over four replicates.

#### Immunohistochemistry

Immunohistochemistry was performed as previously described. Briefly, Ferret lung and human trachea sessions at 5 μm were deparaffinized and rehydrated, followed by an antigen retrieval step by heating the slides for 10 min in sodium citrate. The endogenous peroxidase was inactivated by 1% H2O2 in MEOH. Tissues were blocked by the 3% BSA in PBS overnight at 4°C. The precomplexed HA, strepmab, and goat-anti-human antibodies were incubated with tissues for 90 min at RT. For ferret lung, the HA was 50 μg/mL. For the Human trachea, the HA was 20 μg/ml. After incubation, the tissue was stained by AEC substrate for HRP (Abcam) and subsequently stained by hematoxylin.

#### Hemagglutination assay

The chicken erythrocytes were remodified with different lengths of LacNAc, terminal α2,6 sialic acid described previously.^10^ 20 μg/mL Hemagglutinin was incubated with strepmab and goat-anti-human HRP on ice for 30 min (same ratio as ELISA). Pre-complexation was diluted serially in 2-fold.) 1% Erythrocytes were added and incubated at RT for 3–4 hours.

#### Flow cytometry analysis

10 μg/mL Hemagglutinin was precomplexed with strepmab and goat-anti-human Alexa Fluor 488 in a 1:0.65:0.325 molar ratio on ice for 30 min. The precomplexed proteins were incubated with 50,000 hCK or hCK-B3GNT2 cells for 30 min at 4°C in the dark. Cells were washed with FACS buffer (PBS containing 1% FCS and 2 mM EDTA) and followed by the centrifuge at 300 rcf for 5 min. The cells were fixed with 1% Paraformaldehyde for 10 min at 4°C in the dark. After being washed with FACS buffer, the cells were resuspended in 100 μL of FACS buffer. Flow cytometry was performed using BD FACSCanto II or the CytoFLEX LX (Beckman Coulter). Data was analyzed using FlowJo software. All cells, single cells, and live cells were gated. Mean fluorescence values for triplicates were averaged, and standard deviations were calculated.

#### Molecular dynamics simulations

Monomeric models of HAs from A/SG/16 and its mutants Y159N/T160I and Y159N/T160I/Y195F were generated with AlphaFold2^44^ version 2.3.2 using the following options: *–models-to-relax=all*, *–enable-gpu-relax*, *–db-preset=reduced_dbs*, and *–model-preset=monomer*. For each mutant and wildtype, thirty structures were generated for models 3, 4, and 5 (90 structure predictions per sequence). Predicted structures were ranked according to the pLDDT score, and the highest-scoring structure for each sequence was used as the starting point for extensive conventional molecular dynamics (MD) simulations. Models were cropped to include residues 49 to 278 and have a pLDDT score >95, indicating high prediction confidence.

N158 of the A/SG/16 model was glycosylated with a biantennary symmetric, not core-fucosylated, eptasaccharide, using the GLYCAM Carbohydrate Builder.^46^ For A/SG/16 and its Y159N/T160I/Y195F mutant, initial geometries of the complex with **D** were prepared, generating the coordinates of **D** using the GLYCAM Carbohydrate Builder^46^ followed by manual overlay onto the AlphaFold2 protein model.

MD simulations were carried out with AMBER 24^47^ using the ff14SB force field for the proteins,^48^ the GLYCAM-06j force field for glycans,^49^ and the TIP3P force field for water.^50^ Unbound and bound models were immersed in a water box with a 10 Å buffer of water molecules and neutralized by adding explicit Cl^−^ counterions (Li-Merz 12–6 normal usage set).^51^ A two-stage geometry optimization approach was implemented. The first stage minimizes only the positions of solvent molecules and ions, and the second stage is an unrestrained minimization of all the atoms in the simulation cell. The systems were then heated by incrementing the temperature from 0 to 300 K under a constant pressure of 1 atm and periodic boundary conditions. Harmonic restraints of 10 kcal mol^−1^ Å^−2^ were applied to the solute, and the Andersen temperature coupling scheme was used to control and equalize the temperature.^52,53^ The time step was kept at 1 fs during the heating stage, allowing potential inhomogeneities to self-adjust. Once equilibrated, the system was subjected to a 2 ns constant volume molecular dynamics simulation at 300 K using the SHAKE algorithm and a 2 fs time step.^54^ Long-range electrostatic effects were modeled using the particle mesh Ewald method.^55^ A cutoff of 8 Å was applied to Lennard-Jones interactions. Production was run with the same setup as three independent 400 ns replicas, for a global simulation time of 1.2 μs per system. Analysis was performed with cpptraj.^56^

#### Conservation analysis

We downloaded all available haemagglutinin (HA) sequence data for all influenza A virus subtypes from the Global Initiative on Sharing All Influenza Data (GISAID) (https://gisaid.org/) EpiFlu database.^57^ After removing sequences low-quality sequences (%5> ambiguous nucleotides) or sequences shorter than 800 nucleotides, the remaining sequences were aligned per subtype against the reference used in the universal numbering scheme using MAFFT (version 7.508).^58,59^ The coding sequence was then translated and the mature positions were converted to the corresponding H3 position using the universal numbering scheme. Conservation of amino acid (AA) at H3 position 195 across subtypes were then determined by simple counting.

For a random subsample of the 3 H3 HA sequences per month collected between 2019 and 2024, we constructed a maximum-likelihood using IQ-TREE (version 2.2.0.3)^60^ using a GTR substitution model and converted it into a time-resolved phylogeny using TreeTime (version 0.9.4).^61^ The tree in Figure S3 is visualized using Baltic https://github.com/evogytis/baltic/tree/master.

### QUANTIFICATION AND STATISTICAL ANALYSIS

#### Glycan microarray

The data was processed by removing the highest and lowest values from 4 replicates, total intensities were plotted as means ± SD using Microsoft Excel and visualized with GraphPad Prism 10, which is shown in Figure 2C.

#### Hemagglutination assay

The triplicate data were processed and plotted as means ± SD with GraphPad Prism 10, e.g., the details can be found in Figure 1E.

#### Flow cytometry assay

Data was analyzed using FlowJo software. The mean value and Standard deviation were calculated over triplicate measurements and plotted with GraphPad Prism 10, e.g., the details can be found in Figure 1D.

#### Enzyme-linked immunosorbent assay

Data was analyzed and visualized using GraphPad Prism 10, which is shown in Figures 1B, 2B, 3B and 4A. The analyzed method was determined using one site-total.

## Supplementary Material

1

Supplemental information can be found online at https://doi.org/10.1016/j.celrep.2025.116007.

## Figures and Tables

**KEY RESOURCES TABLE:** 

REAGENT or RESOURCE	SOURCE	IDENTIFIER

Antibodies		

Strepmab	This paper	N/A
Goat-anti-human IgG antibodies	Invitrogen	Cat#31410; RRID:AB_228269
Goat anti-human-Alexa 555	Invitrogen	A21433; RRID:AB_1500626
Goat anti-human-Alexa 488	Invitrogen	A11013; RRID:AB_2534080
Streptavidin-HRP	Biolegend	Cat# 405210

Bacterial and virus strains		

MC1061 competent cells	This paper	N/A

Biological samples		

β3GnT2	Brosziet, F. et al.^43^	N/A
B4GalT1	Brosziet, F. et al.^43^	N/A
ST6Gal1	Brosziet, F. et al.^43^	N/A
Calf intestine alkaline phosphatase	NEB	NEB M0525S
*Erythrina cristagalli* agglutinin	Vector Labs	Cat # B-1145
*Sambuca nigra* agglutinin	Vector Labs	Cat # B-1305
*Maackia amurensis*	Vector Labs	Cat # B-1315

Chemicals, peptides, and recombinant proteins		

symmetrical bi-antennary N-glycan with a mono-LacNAc **A**	Brosziet, F. et al.^43^	N/A
symmetrical bi-antennary N-glycan with a di-LacNAc **C**	Brosziet, F. et al.^43^	N/A
symmetrical bi-antennary N-glycan with a tri-LacNAc **E**	Brosziet, F. et al.^43^	N/A
Asymmetrical N-glycan with a di-LacNAc **B**	Brosziet, F. et al.^43^	N/A
asymmetrical N-glycan with a tri-LacNAc **D**	Brosziet, F. et al.^43^	N/A
A/Switzerland/9715293/2013 wt recombinant	Spruit, C. M. et al.^28^	N/A
A/Switzerland/9715293/2013 mutations recombinant	This paper	N/A
A/Singapore/INFIMH-16–0019/2016 wt recombinant	Unione, L. et al.^17^	N/A
A/Singapore/INFIMH-16–0019/2016 mutations recombinant	This paper	N/A
A/Darwin/9/21 wt recombinant	This paper	N/A
A/Netherlands/00010/2019 wt recombinant	Spruit, C. M. et al.^28^	N/A
A/Hongkong/1/1968 wt recombinant	Spruit, C. M. et al.^28^	N/A
UDP-Gal	Roche	Cat #07703562103
UDP-GlcNAc	Roche	Cat # 11787900103
CMP-Sialic	Roche	Cat # 05974003103

Deposited data		

A/Darwin/9/21 HA Amino acids	NCBI	WEY08928.1
The script for carbohydrate-microarray-processing	Broszeit, F.et al.^10^ Zenodo	https://doi.org/10.5281/zenodo.5146251

Experimental models: Cell lines		

HEK293S GnTI(-)	ATCC	CRL-3022
hCK	Spruit, C. M. et al.^28^	N/A
hCK-B3GNT2	Spruit, C. M. et al.^28^	N/A

Experimental models: Organisms/strains		

Ferret Lung	This paper	N/A
Human trachea	This paper	N/A

Oligonucleotides		

available upon request	This paper	N/A

Recombinant DNA		

A/Switzerland/9715293/2013 mutations	This paper	N/A
A/Singapore/INFIMH-16–0019/2016 mutations	This paper	N/A
A/Darwin/9/21 wt	This paper	N/A

Software and algorithms		

AlphaFold2 (version 2.3.2)	Jumper et al.^44^	https://github.com/google-deepmind/alphafold
Prism (V10)	GraphPad Software	https://www.graphpad.com/
GLYCAM Carbohydrate Builder	NIH	https://glycam.org/
AMBER 24	The amber project	https://ambermd.org/AmberMD.php
cpptraj	The amber project	https://ambermd.org/tutorials/analysis/tutorial0/index.php
MAFFT (version 7.508)	MAFFT	https://mafft.cbrc.jp/alignment/server/index.html
IQ-TREE (version 2.2.0.3)	IQ-TREE	http://www.iqtree.org/

Other		

Regenerated Cellulose Membrane Filter, 30 kDa NMWCO	Merck	PLTK06210

## Highlights

- The amino acid Y159N/T160I abrogates binding and is rescued by Y195F
- There is an epistatic effect in 130-loop and 190-helix during evolution
- Asymmetrical α2,6-sialylated tri-LacNAc binding correlates with respiratory tissue binding
