# Supplementary material for: Epistasis in the receptor-binding domain of contemporary H3N2 viruses that reverted to bind sialylated di-LacNAc repeats
Source: Cell Rep. Author manuscript; Available in PMC 2025 Sep 16. (PMC12439434; doi:10.1016/j.celrep.2025.116007)
Supplement: 1 [file NIHMS2107242-supplement-1.pdf]

Cell Reports, Volume *44*

Supplemental information

Epistasis in the receptor-binding domain of contemporary H3N2 viruses that reverted to bind sialylated di-LacNAc repeats

Ruonan Liang, Francesca Peccati, Niels L.D. Ponse, Elif Uslu, Annelies J.H. de Rooij, Alvin X. Han, Geert-Jan Boons, Luca Unione, and Robert P. de Vries

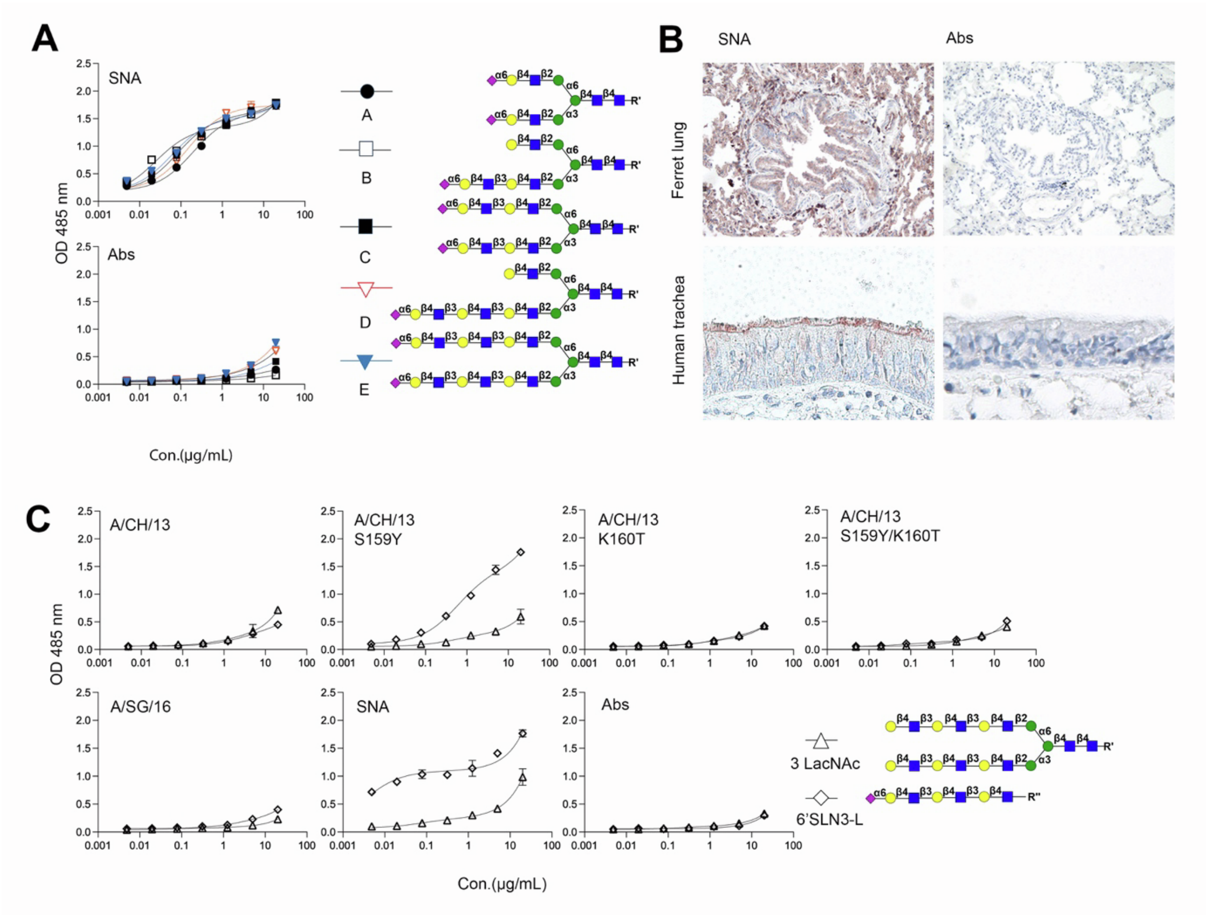

Figure S1. The correlation between glycan lengths with HA Y159/T160. A. Binding avidities SNA and antibodies only (Abs) were measured by ELISA. **B.** Tissue staining of SNA and Abs to ferret lung and human trachea. **C.** Binding avidities to 6' SLN3-L and 3LacNac without sialic acid for A/CH/13 WT and mutants, A/SG/16, SNA, and Abs.

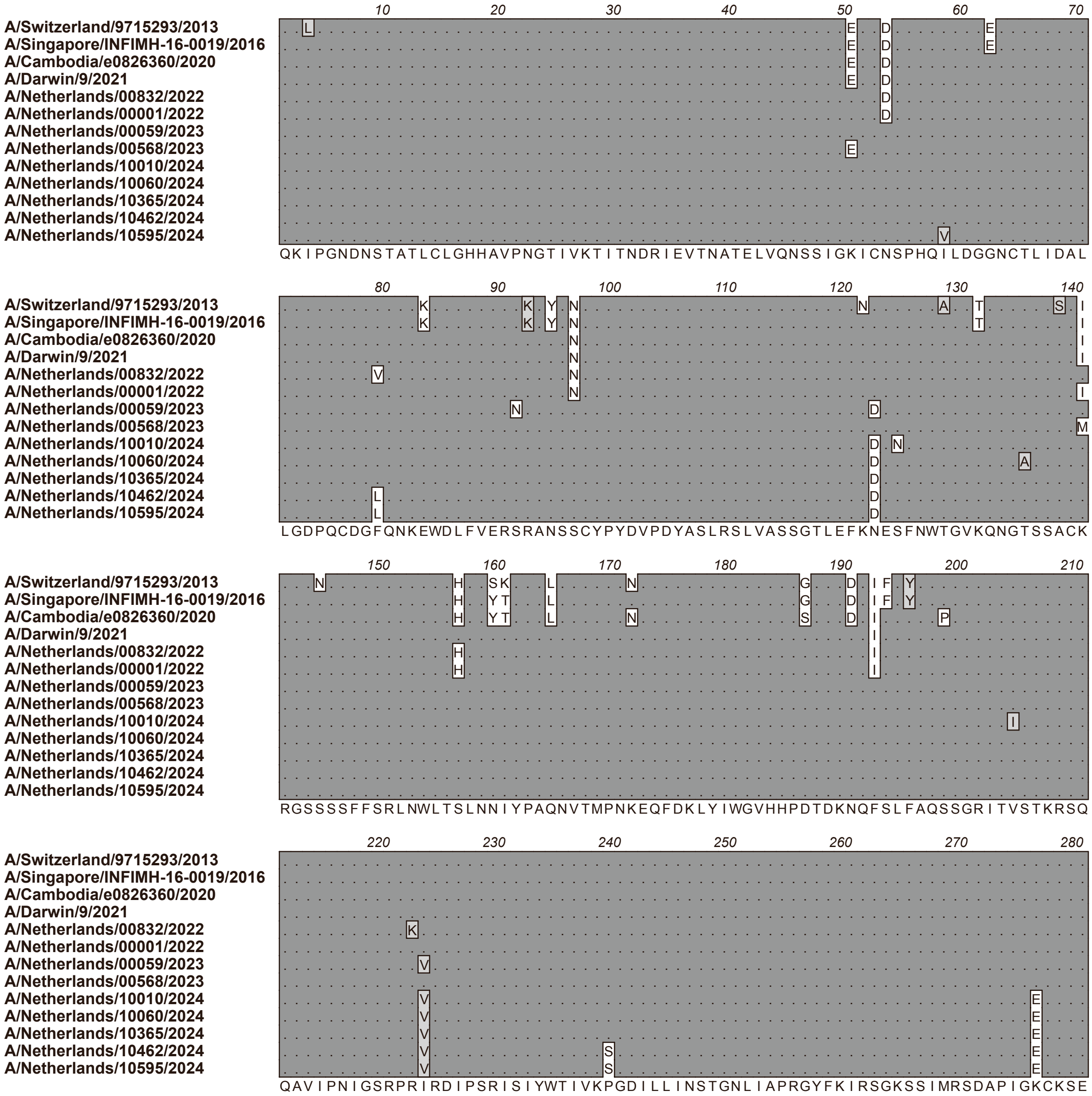

Figure S2. amino acid alignment of HA1 (1-280) comparing vaccine strains with recent circulating H3N2 viruses.

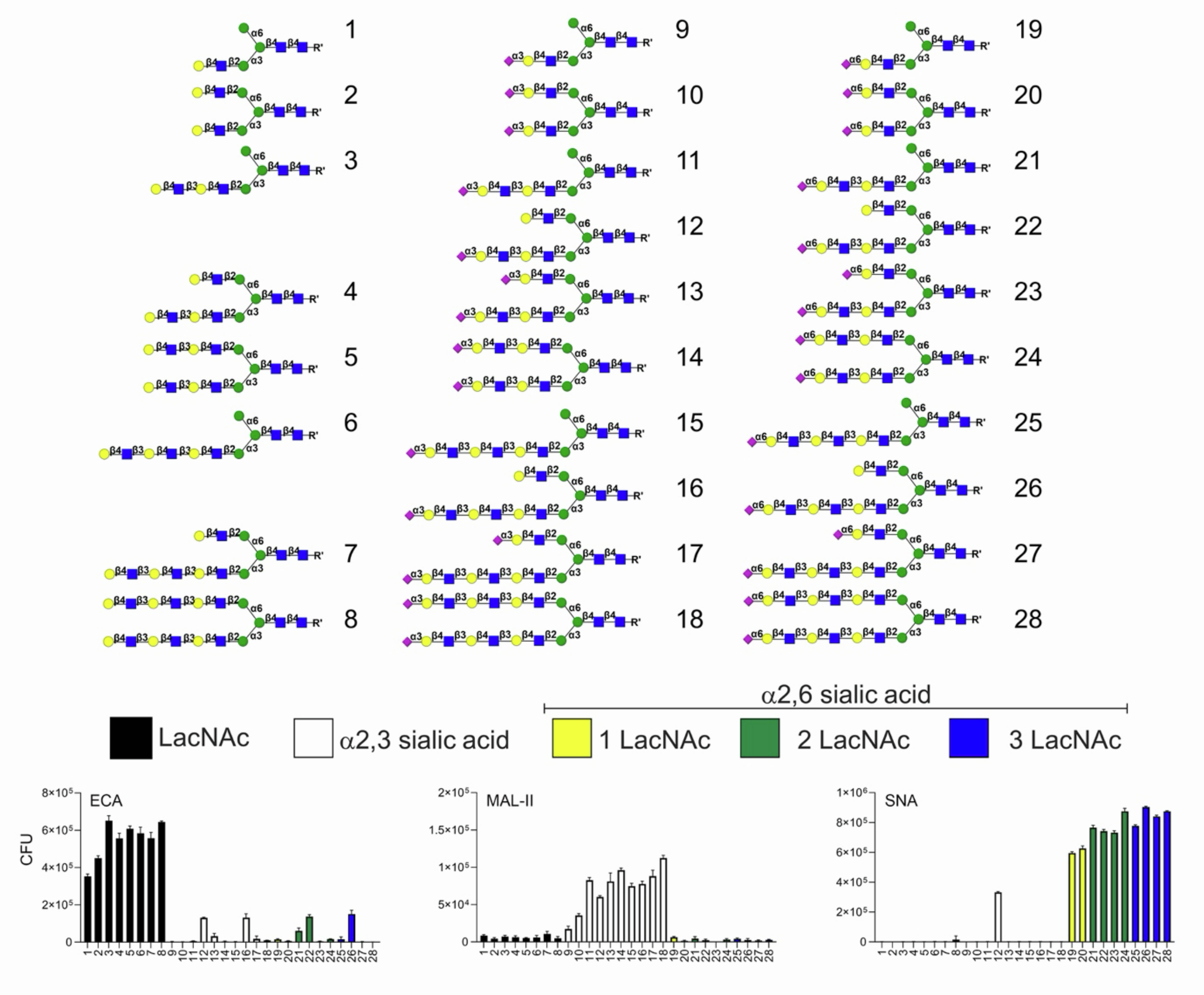

Figure S3. Population of the the glycan array and quality control thereof. ECA recognizes terminal galactose (**#1-8, 12 ,16, 22** and **26**). MAL-II recognizes the glycans terminating with α2,3 sialic acid **(#9-18**). SNA recognizes the glycans terminating with α2,6 sialic acid (**#19-28**).

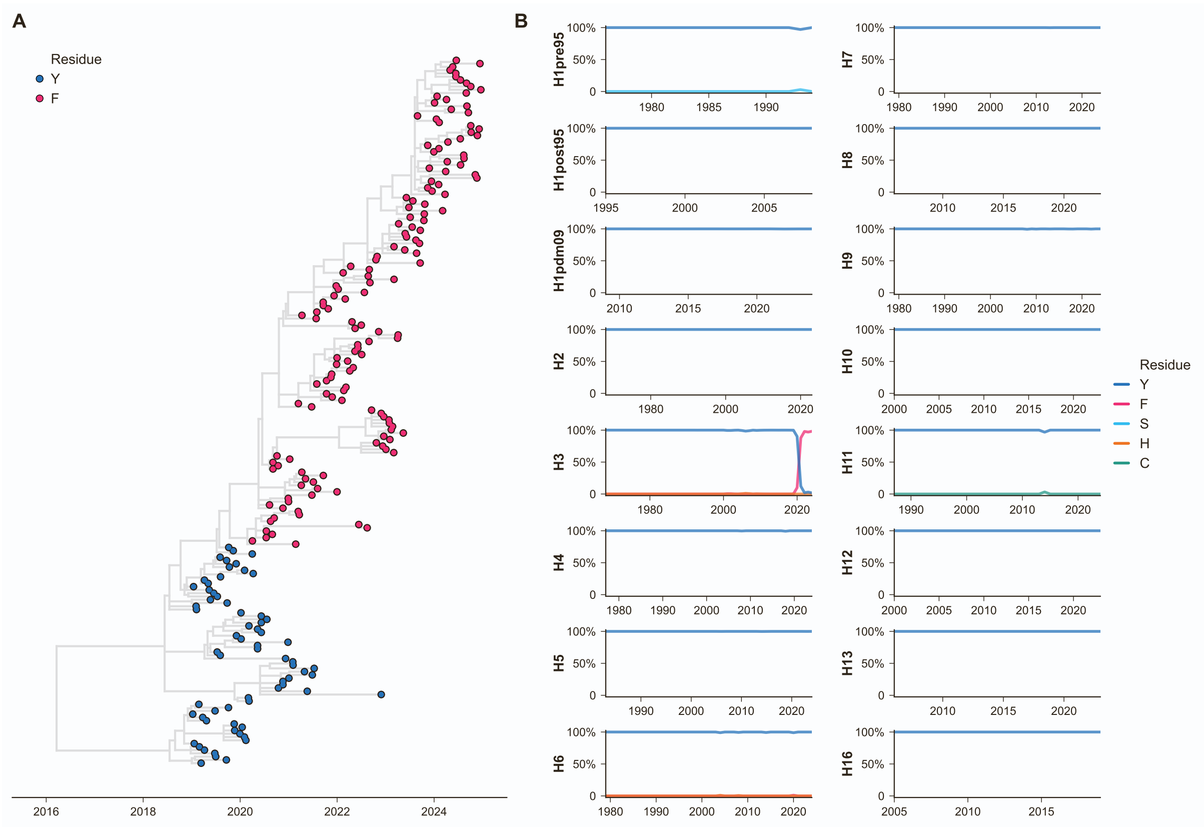

Figure S4. Conservation of tyrosine (Y) at position 195 (H3 numbering) in haemagglutinin (HA) across influenza A virus subtypes (IAV). A. Time-resolved phylogenetic maximum-likelihood tree of H3 HA sequence collected between 2019-2024. Tips are colored by the amino acid present at position 195. Pink tips indicate tyrosine at position 195, blue tips indicate phenylalanine (F). **B.** Amino acid frequency trajectories at position 195 for all IAV subtypes. H14 (n=61over 12 years), H15 (n=24 over 6 years), H17 (n=3), and H18 (n=1) are not included in the figures as the number of sequences over time was too small to plot reliable frequency trajectories.

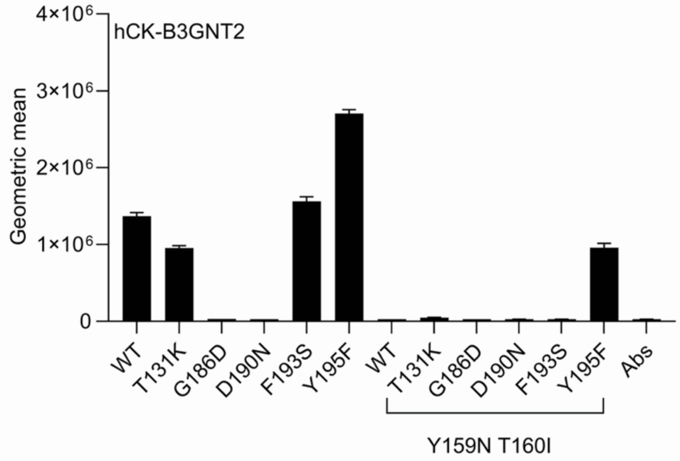

Figure S5. Flow cytometry analysis of binding to hCK-BGNT2 cells.

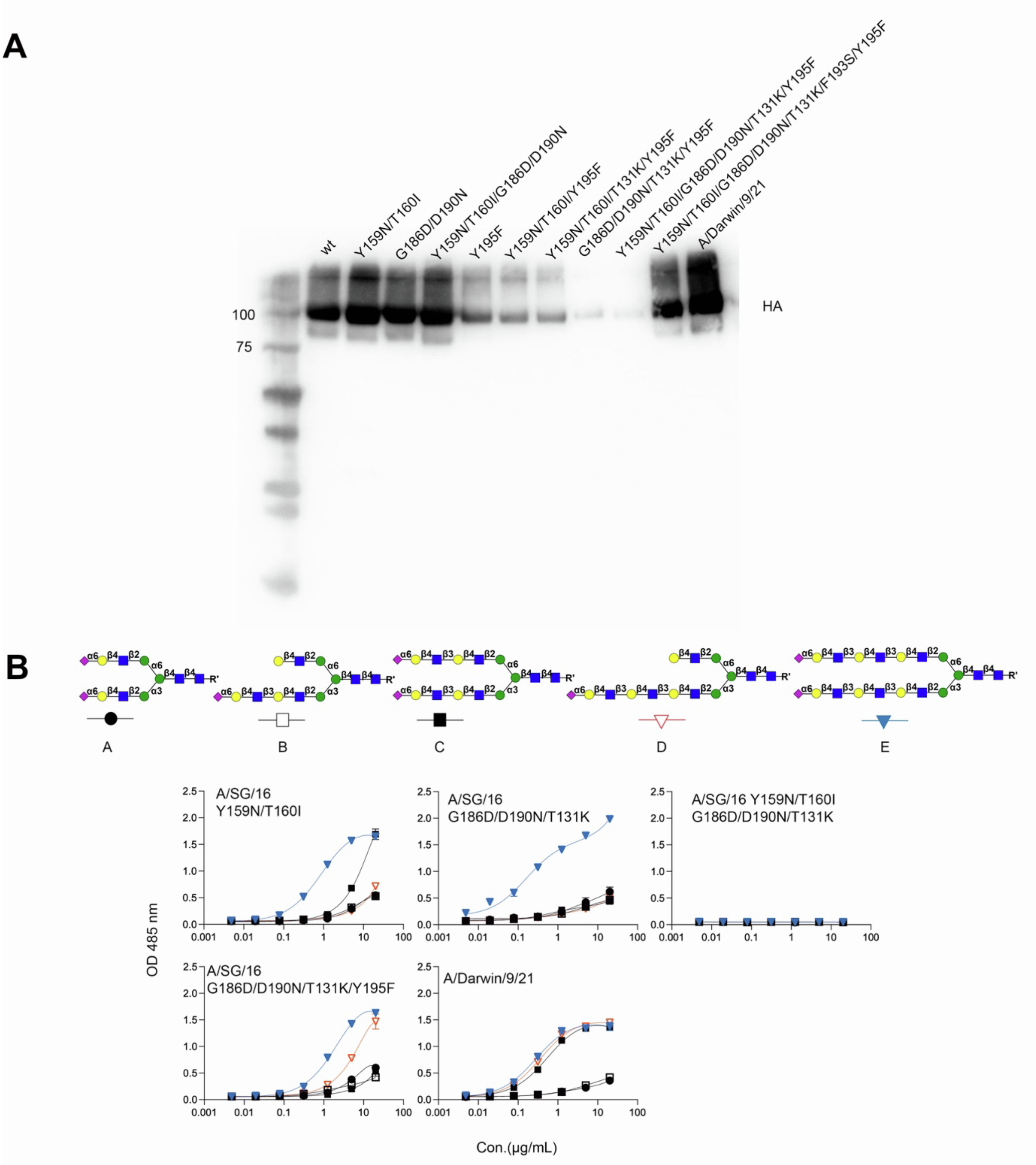

Figure S6. The binding activity of different combinations of mutations. A. Western blotting of HEK293S GnTI cell supernatants transfected with plasmids containing different mutations of A/SG/16 with or without Y195F. **B.** Binding activity of A/SG/16 mutants and A/Darwin/9/21 HA by ELISA.

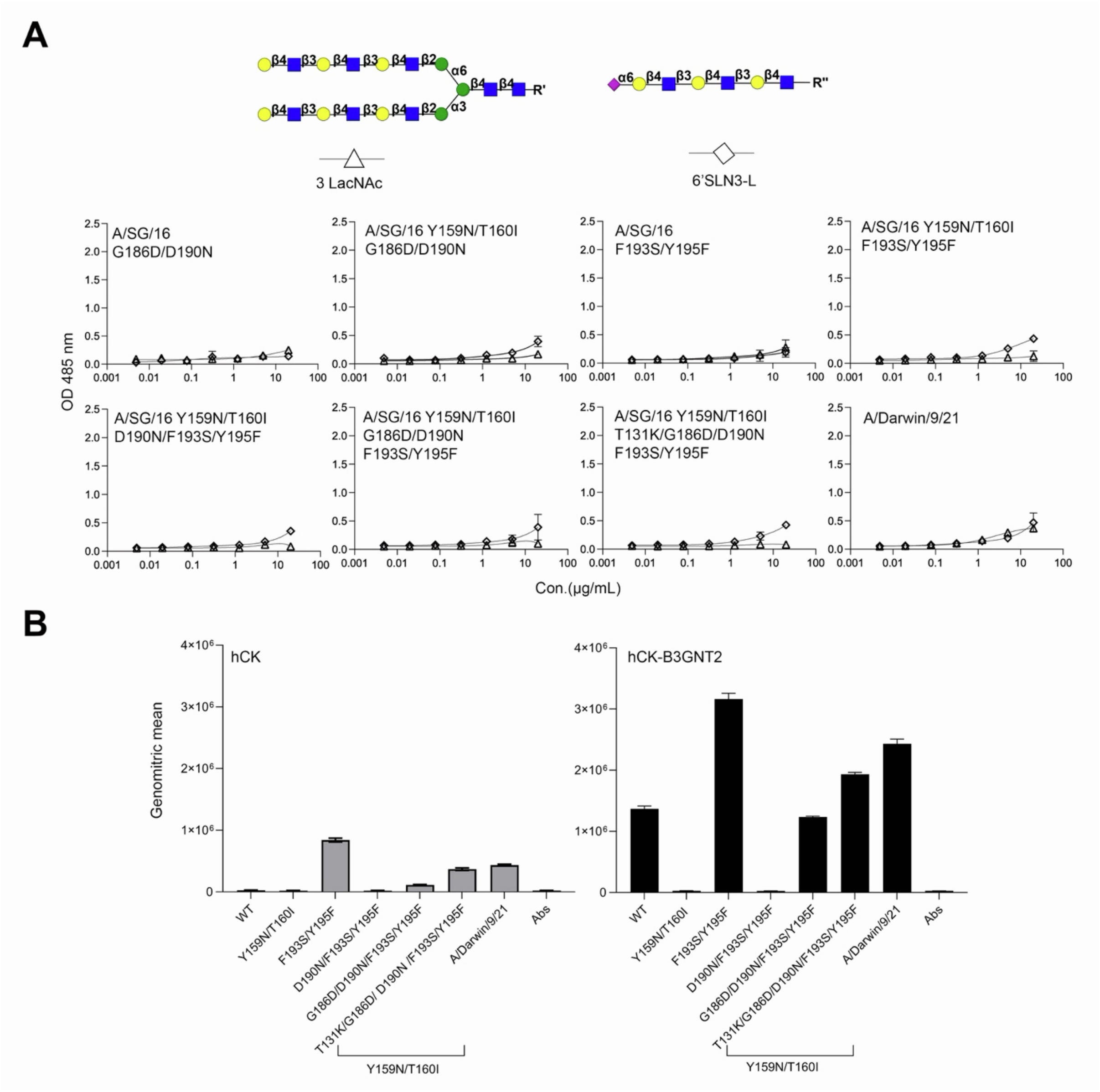

Figure. S7. Binding activity of different mutant based on the background of A/SG/16 or A/SG16 Y159N/T160I. A. Binding avidities to 6’ SLN3-L and 3LacNAc without sialic acid. **B**. Flow cytometry analysis of binding to hCK and hCK-BGNT2 cells.

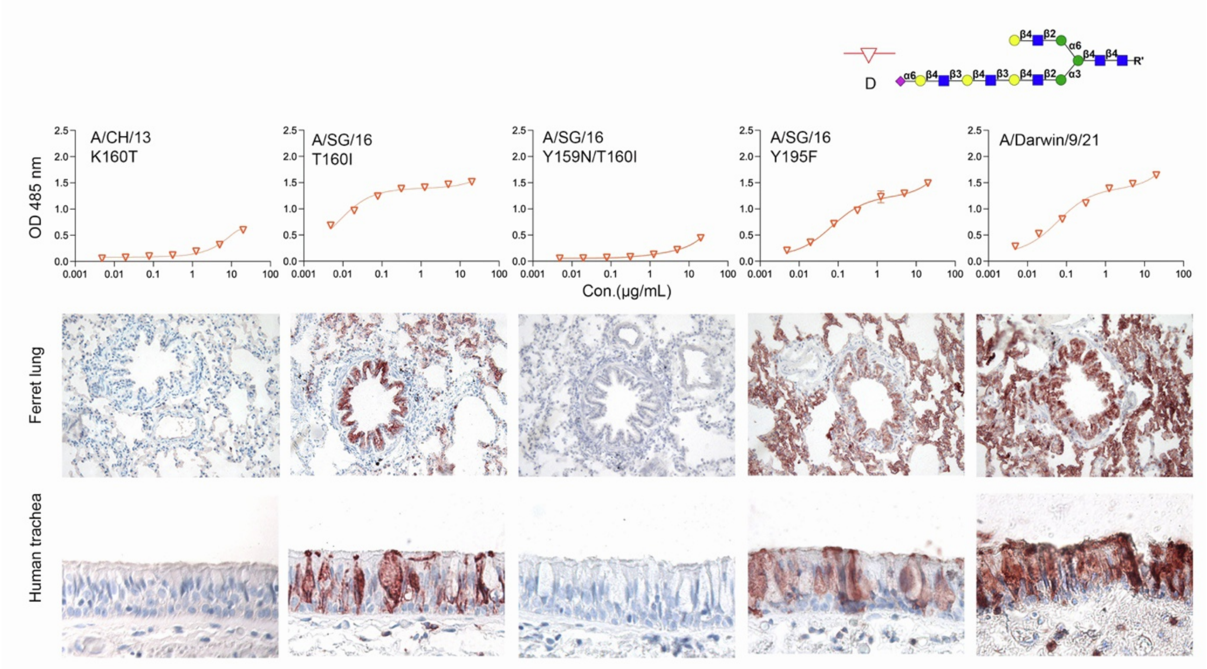

Figure S8. The relationship between binding to asymmetrical N-glycan with a α2,6 sialylated triLacNAc (compound D) and binding to human and ferret respiratory tissues. ELISA result (up) *vs* Tissue staining (down).
